# Development and implementation of a comprehensive ultrasound curriculum for undergraduate medical students – a feasibility study

**DOI:** 10.1186/s12909-019-1611-1

**Published:** 2019-05-28

**Authors:** Nora Celebi, Jan Griewatz, Nisar P. Malek, Sarah Krieg, Toni Kuehnl, Reinhold Muller, Jan Pauluschke-Fröhlich, Ines Debove, Reimer Riessen, Stephan Zipfel, Eckhart Fröhlich

**Affiliations:** 1 PHV Dialysis Center Waiblingen, Beinsteiner Straße 8/3, 71334 Waiblingen, Germany; 2 Eberhard-Karls University, Competence Centre for University Teaching in Medicine, Baden-Wuerttemberg, Elfriede-Aulhorn-Str. 10, D-72076 Tuebingen, Germany; 3 Department of Internal Medicine I (Gastroenterology, Hepatology, Infectious Diseases), University Hospital Tübingen, Otfried-Müller-Str. 10, 72076 Tübingen, Germany; 4 Medical Faculty, Eberhard-Karls University, Geissweg 5/3, 72076 Tübingen, Germany; 5 Professorial Research Fellow AITHM, James Cook University, 1/14-88 McGregor Rd, Smithfield, QLD 4878 Australia; 6 Department of Gynecology, University Hospital Tübingen, Calwerstraße 7, 72076 Tübingen, Germany; 7 Department of Neurology, Inselspital, Freiburgstrasse 18, 3010 Bern, Switzerland; 8 Department of Internal Medicine, Intensive Care Unit, University Hospital Tübingen, Otfried-Müller-Str. 10, 72076 Tübingen, Germany; 9 Department of Internal Medicine VI (Psychosomatic medicine), University Hospital Tübingen, Osianderstr. 5, 72076 Tübingen, Germany

**Keywords:** Medical student, Sonography, Ultrasound, Undergraduate, Peer teaching, Implementation, Curriculum, Student tutor

## Abstract

**Background:**

Ultrasound is one of the most important imaging techniques in clinical medicine with unique advantages. Skills in ultrasound imaging are very usefull for physicians including novices and thus also mandated by the Task Force “National Competence-Based Learning Objectives for Undergraduate Medical Education” (NKLM) in Germany and as well as by the German Ultrasound Society (Deutsche Gesellschaft für Ultraschall in der Medizin, DEGUM). Since ultrasound is best learned hands-on in very small supervised groups, we developed and implemented a comprehensive ultrasound-curriculum for all undergraduate medical students of our faculty using a peer-teaching concept.

**Methods:**

We used Kern‘s six-step model of curricular development comprising (1) problem identification and general needs assessment, (2) needs assessment of the targeted learners, (3) goals and objectives, (4) educational stategies, (5) implementation, and (6) evaluation and feedback.

**Results:**

The developed curriculum covers basic ultrasound of the abdomen and the throat, eFAST (Extended Focused Assessment with Sonography for Trauma), lung-ultrasound, FEEL (Focused Echocardiography in Emergency Life Support) and compression duplex sonography of the thigh deep vein system. All 5th year medical students receive a 90 min lecture on ultrasound basics by a faculty member and then a 12.5 h hands-on course divided into three sessions with one student tutor for every 4 students. The students are provided with a script (PDF-File) that covers all the learning goals, including example images of pathologies. The student tutors are trained during a 1 week ultrasound course and a 21-day rotation through seven different ultrasound laboratories. In addition, they undergo a standardized 1.5 day didactical training. Prior to the implementation for all students, the overall course was tested on 27 volunteer students. These students rated (on a 6-point Likert scale from 1 = excellent to 6 = very poor) the satisfaction with the student tutors and the faculty members as 1.4 ± .9 (mean ± stddev) and 1.3 ± .5 respectively.

**Conclusion:**

A comprehensive ultrasound curriculum for all undergraduate medical students using a peer-teaching concept is feasible. Further studies are needed to evaluate in detail the learning outcomes for students and student tutors.

## Background

As an imaging technique ultrasound has several unique features. It is portable, does not involve radiation, is inexpensive, can be repeated multiple times, shows the images in real-time, facilitates urgent medical decision making and the basic technique is comparably easy to learn ([24]; “Statement from the DEGUM board regarding the editorial entitled “Who’s Doing Your Scan? A European Perspective on Ultrasound Services.”,” 2018). Ultrasound skills have the potential to increase the information gathered from physical examination and to greatly improve the physician’s ability to care for – especially critically ill - patients [9, 14, 19, 20, 27].

The system for the provision of ultrasound examinations differs between health systems, especially between anglo-american countries and mainland Europe [28]). For example in Great Britain about 80% of the examinations are performed by especially trained sonographers, 19% by radiologists and only 1% by physicians of other specialties [28]. In countries like Germany, Austria oder Switzerland, ultrasound examinations are predominantly performed by the treating physician of the corresponding medical discipline, such as internists, gynecologists, fetal medicine specialists, surgeons, urologists, neurologists, etc. and considered an integral part of the specialist training and daily care of patients [26, 28]. To ensure the quality in the provision of ultrasound examinations, scientific societies – as for instance in Germany the DEGUM (Deutsche Gesellschaft für Ultraschall in der Medizin) - provide training and certification of ultrasound providers, advance ultrasound techiques and also conduct the scientific evaluation of these techniques.

Recognizing the importance of ultrasound, the Task Force “National Competence-Based Learning Objectives for Undergraduate Medical Education” (NKLM) in Germany mandates the incorporation of ultrasound into the undergraduate curriculum for all medical students [25]. There have been several attempts to teach ultrasound skills to undergraduate medical students, usually for a limited number of students and on circumscribed topics only [16, 29]. On the other hand, Baltarowich et al. proposed a comprehensive list of learning goals for undergraduate students that is so extensive, that even the authors state that it is not likely that any faculty will be able to implement them all [3]. Thus, the question seems not, whether to teach ultrasound to all undergraduate students, but what should be taught and how it should be optimally delivered.

Several aspects have to be considered in the development of an ultrasound curriculum. Given the ever increasing amount of knowledge and number of skills medical students have to tackle during their undergraduate education, a careful selection of the learning goals is warranted [5]. A successful ultrasound course should not only sample a wide range of ultrasound techniques but also teach the students under which circumstances ultrasound might be the appropriate imaging technique. Performing an ultrasound examination involves theoretical knowledge about the physics of ultrasound and anatomy, pattern recognition for pathologies, as well as the ability to use the device correctly to obtain meaningful images. While the theoretical parts can be efficiently taught in lectures or by the means of scripts, the actual scanning is best taught hands-on in very small supervised groups. Thus, the range of skills that can be taught and the numbers of students is limited by the number of available instructors [7, 17, 29, 32]. Given this situation, the employment of a peer teaching concept seems appropriate. Several studies have already shown that medical students can aquire in a few weeks enough knowledge and skills to teach simpler ultrasound skills as effectively as faculty members [1, 8, 18]. However, this seems not to be the case for more complex and advanced echocardiography skills [21].

While ultrasound can be useful in almost every clinical discipline, not all situations are time-critical and thus in some disciplines, the novice ultrasound learner can wait for an experienced examiner to instruct him. Thus, in our opinion, undergraduate ultrasound education should predominantly focus on time-critical emergency ultrasound skills, which enable the novice physician to gather relevant information without the need to wait for a more experienced supervisor.

We therefore aimed to develop a comprehensive curriculum for a limited number of essential skills that satisfy the demands of the NKLM, are easy to learn for both the student tutors and the tutees, benefit the patients most, and can be implemented and sustained for all students.

## Methods

### Problem identification and general needs assessment

In order to determine what best to include into an ultrasound curriculum for undergraduate students, in June 2016 we consulted three main sources:

- The German ultrasound society (Deutsche Gesellschaft für Ultraschall in der Medizin, DEGUM) with the task force undergraduate education, which proposes content for an undergraduate ultrasound curriculum and the task force emergency sonography, which developed a curriculum for ultrasound skills that novice physicians should ideally already have mastered when taking on responsibilities in the care of emergency patients;
- The Task Force “National Competence-Based Learning Objectives for Undergraduate Medical Education” (NKLM) in Germany (section diagnostic skills); and
- The published literature to research ultrasound skills or algorithms that are easy to learn and have a high impact in clinical practice, and to identify any potential ultrasound curricula already proposed for undergraduate students.

### Needs assessment of the targeted learners

The ultrasound examination techniques identified by the above described needs assessment were then discussed extensively with faculty members of the different subspecialties which provide the various examinations as well as with medical students in different stages of their clinical training (3rd to 6th year).

Subsequently, targeted learning objectives already included in existing curricula were eliminated.

### Goals and objectives

Finally, for each identified ultrasound technique, technical, physiological and pathological properties were operationalized in August 2016. Again, faculty members of the providing specialties and medical students were heavily involved in the process.

### Educational stategies

Since the learning goals comprised theoretical knowledge as well as practical skills, different educational strategies were blended to cover all areas and aspects. We employed the techniques developed, used and evaluated by the DEGUM for ultrasound training: scripts and lectures for the theoretical knowledge and supervised hands-on training fot the practical scanning.

In addition, the training curriculum for the student tutors who oversee the practical ultrasound training was developed in October 2016. Both curricula are detailed in the following results section.

### Education of the student tutors

The student tutors generally have the same learning goals as the medical students, however their education is extended and additionally covers didactical content. The education was developed in close discussion with the ultrasound student tutors and –tutees.

### Implementation

Initially, the office of the dean of student affairs and the advisory board of study affairs, (comprising both faculty members and medical students) were contacted to obtain permission and to secure support and funding for the student tutor project. A budget to cover the expenses for the student tutor training was granted and we thus could approach all ultrasound stakeholders with the permission of the dean of student’s affairs to proceed with the course.

For the implementation, a suitable slot in the curriculum was chosen and all departments that participated in the curriculum and in the training of the student tutors we involved. Subsequently the infrastructure necessary to sustain the organization of the ultrasound curriculum was established.

With the first batches of trained tutors the curriculum was tested as an elective course on 27 students (3rd to 5th year) in November 2016 and January 2017 before its mandatory implemention for all students. A total of 21 tutees were asked to rate their tutor on a global 6-point Likert scale with 1 = excellent, 6 = very poor. As a comparison, 6 students were taught by faculty members. The data were recorded in Microsoft Excel 2010, Redmont, USA. Data are displayed as mean and standard deviation.

### Evaluation and feedback

The curriculum was incorporated into the mandatory internship Internal Medicine starting October 2017 which has a marked final examination as required by the German Medical Licensure Act. So the students undergo a theoretical examination (multiple choice questions) and an OSCE (objective structured clinical examination) to demonstrate their knowledge and their skills. The satisfaction of the students with the curriculum is assessed with the mandatory online evaluation every term.

### Statistics

Statistical analyses were conducted using the software package SPSS Version 25. Since the underlying distributions proved to be normal, means and standard deviations were used for descriptive purposes.

## Results

### Problem identification and general needs assessment

The DEGUM section ultrasound for undergraduate students and the section emergency ultrasound for novice physicians of the German ultrasound society proposed the following topics to be included into the curriculum [11, 10]:

- Focused abdominal ultrasound with gallbladder, kidneys, bladder, aorta and vena cava
- Ultrasound guided puncture
- eFAST (extended focussed assessment with sonography for trauma)
- Duplex sonography of the deep veins of the thigh
- Thoracic ultrasound

The Task Force National Competence-Based Learning Objectives for Undergraduate Medical Education (NKLM) states the following learning objectives [25]:

- Indicate appropriate ultrasound examinations (ID 15.3.1)
- Interpret images in order to form a diagnosis (ID 15.3.1.2)

In the literature there is extensive support for eFAST, FEEL (Focused Echocardiography in Emergency Life Support) and thoracic ultrasound to aide the care for critically ill patients [14, 22, 23, 30, 31]. The European Federation of Societies for Ultrasound in Medicine and Biology (EFSUMB) recommends a pre-clinical course to enhance student understanding of anatomy, physiology and pathology utilizing ultrasound and a clinical course which is supposed to teach students how to use ultrasound effectively as a problem-solving tool in the diagnosis of disease, recommending the combination of lectures and a peer-teaching concept [6]. Several other proposed curriculums recommend similar content [2, 3, 14, 32].

### Needs assessment of the targeted learners

Prior to the implementation of our curriculum, ultrasound guided puncture was already covered in a different practical course, so this topic was not included in our curriculum. Apart from that, only a peer-teaching abdominal and neck ultrasound course was offered to all 5th year medical students. Other topics were not routinely taught. Our curriculum replaced the abdominal and neck ultrasound course.

### Goals and objectives

The needs assessment process resulted in identifiying the following goals and objectives: The successful student will be able to …

1. Describe the origin of artifacts like reverberation, dorsal acoustic enhancement, dorsal acoustic shadowing, and cystic border shadowing and how they affect the ultrasound image.
2. Use Gain and Depth to optimize imaging.
3. Denominate for every probe position, where on the monitor is left, right, cranial, caudal, ventral or dorsal.
4. Depict liver, common bile duct and gallbladder, aorta, celiac trunc, upper mesenteric artery, renal arteries, inferior vena cava, renal veins, splenic vein, portal vein, confluens vena portae and explain the topographic relationships between the vessels. Depict pancreas and spleen. Depict Morrison’s, Koller’s and Dougla's' pouch. Depict kidneys, ulterus, vagina, prostata and seminal vesicle. Denominate within the kidneys parenchyma, pyelon and mark pyramids.
5. Perform a complete abdominal scan in the described sequence fluently and denominate the sections and the marked anatomic structures.
6. Describe how to recognize the following pathologies: steatosis hepatis, advanced liver cirrhosis, liver cysts, liver hemangiomas, liver metastasis; intra- and extrahepatic cholestasis, gallstones, sludge in the gall bladder, cholecystitis, gall polyps; a collaptic or congested inferior vena cava, aortic aneurysm, enlarged paraaortal lymphnodes; pancreatitis, pancreatic pseudocysts, pancreatic tumors; enlarged spleen; ascites; kidney cysts and masses, obstruction of the urether; foreign bodies in the bladder, masses in the bladder, enlarged prostate, myoma of the uterus.
7. Depict the heart in the parasternal long axis, the parasternal short axis, and the apical four chamber view from subxiphoidal.
8. Denominate left and right atrium, left and right ventricle, septum, aortic-, mitral- and tricuspid valve, aortic root, papillary muscle and pericard in in the aforementioned views.
9. List how to recognize pericardial effusion, a severely impaired left ventricular function, right heart failure, volume depletion, gross valvular dysfunction or a true pulseless electrical activity
10. Depict thyroid, internal jugular vein, and the carotid arteries.
11. Explain how to recognize thyroid nodules, thyreoiditis or enlarged lymph nodes.
12. Explain, how the doppler-effect is used to estimate flow velocities, and list criteria how to optimize the duplex image.
13. Demonstrate compression imaging of the deep veins of the thighs and explain, how a deep vein thrombosis is excluded.
14. Depict ribs, visceral and parietal pleura and diaphragm.
15. Explain how to recognize a pleural effusion, a pneumothorax, pulmonary edema or a lung consolidation.
16. Demonstrate the FAST positions 1–5.
17. Explain how to recognize free fluids in these positions.

### Educational strategies

The theoretical knowledge is conveyed in a 90-min lecture for all medical students performed by a faculty member in the first week of the term. In addition, all learning goals are covered in a script that is handed to the medical students. All pathologies are represented with image examples.

The practical hands-on part is delivered to the students by means of a 12.5-h tutorial divided into three sessions with a ratio of one student tutor for every four medical students.

### Education of the student tutors

The student tutors generally have the same learning goals like the medical students; however, their education is extended and covers didactical content as well. The training took 36 days.

In the first week of the term break, a five-day ultrasound course for the new student tutors held by faculty members takes place. This course starts with an anonymous written test of the students’ already existing anatomical / ultrasound knowledge. The actual content of the course is then delivered by alternating for each section between theoretical lectures and hands-on practicals. In summary, day 1 covers the basic physics and the handling of the ultrasound device as well as image optimisation. The anatomical focus is then on the liver, gallbladder, and bile ducts. Day 2 deals with the retroperitoneum, abdominal vessels, lymphnodes, pancreas, spleen, kidneys, bladder, uterus, and prostate covering their anatomy, scanning techniques as well as pathologies. Day 3 summarizes the above described skills to achieve a systematic examination of the abdomen. In a second section, focused echocardiography in emergency life support including thorax and heart (FEEL) is taught. Day 4 deals with the thyroid, jugular veins, carotid arteries, and lymph nodes - again including their anatomy, scanning technique, and pathologies. In the same way, duplex sonography and compression sonography of the deep veins are detailed. Day 5 deals specifically with thorax examination and an extended focus on ultrasound in trauma assessement (eFAST). For the eFAST, students could practice on an ultrasound dummy. The course concludes with theoretical and practical post-test (including multiple choice tests and objective structured clinical examinations), the course evaluation, and a debriefing.

Subsequently the student tutors rotate through and sit in on seven different ultrasound laboratories (including gastroenterology, oncology, endocrinology, nephrology, echocardiography and intensive care unit) to practice their scanning technique and get familiar with pathologies. The rotation can be attested as a clinical elective.

In addition, the student tutors attend a 1.5 day didactical training comprising 12 units on didactical strategies including role and responsibilities of a student tutor; creating an environment that supports learning; reflection and feedback; explaining and visualizing; activating teaching strategies; conveying practical skills; handling difficult teaching situations; and simulation of a teaching situation with video-feedback.

### Implementation

With the first batches of trained tutors, the curriculum as an elective course was tested on 27 students (3rd to 5th year) before implementing it mandatory for all students. A total of 21 tutees were asked to rate their tutor on a global 6-point Likert scale with 1 = excellent, 6 = very poor. As a comparison, 6 students were taught by faculty members. These students rated the satisfaction with the student tutors and the faculty members as 1.4 ± .9 (mean ± stddev) and 1.3 ± .5 respectively.

Involving the respective departments that participated in the curriculum and in the training of the student tutors, a suitable slot for the implementation of the project in the curriculum was identified. Then the infrastructure to sustain the organization of the ultrasound curriculum was created.

The ultrasound curriculum was embedded into the mandatory internship Internal Medicine in the fifth year right before the practical and final sixth year of the undergraduate medical school. A fifth year cohort comprises usually about 160 students per term. The hands-on part is held within a week, so that in a 14-week term all students can be taught in batches of about 12 students. Our ultrasound training facility „DocLab "has four ultrasound devices dedicated only for student education.

For the hands-on part of the ultrasound curriculum, one student tutor attends to four tutees, so we estimate that a total of 525 student tutor hours per term are required. Usually, a student tutor teaches about 10–20 h per term, thus a pool of 40 student tutors is necessary to staff the hands-on ultrasound course. The number of student tutors is limited by the capacity of the ultrasound laboratories to train them, but depending on the length of the term break 10–16 new student tutors can be trained each term which is sufficient to maintain the pool of ultrasound tutors when recruited mainly from third and fourth year.

The wages of the student tutors are covered by the student tutor budget of the office of the dean of student affairs. The student tutor training can be attested as a clinical elective, but as an additional incentive student tutors can earn an official certificate with the seal of the DEGUM when having completed at least three complete tutorials. Moreover, a specific working group has been established as a long-term support for this tutor-based project.

### Evaluation and feedback

The skills and knowledge of the student tutors is assessed during the ultrasound course for new student tutors at the beginning of the term break.

Since the internship Internal Medicine is required to have a marked final examination by the German Medical Licensure Act, the content of the ultrasound curriculum is incorporated into the examination with both a theoretical examination (multiple choice questions) and an OSCE (objective structured clinical examination). The satisfaction of the students with the course is assessed with the mandatory online evaluation every term.

## Discussion

Using Kern’s six step approach [12], we developed a comprehensive ultrasound curriculum for undergraduate medical students that fulfills the requirements of the NKLM, the recommendations of the DEGUM and can be implemented and sustained for all students.

There are several factors to be considered when implementing an ultrasound curriculum. First and foremost, knowledge about ultrasound physics, anatomy and pathologies is insufficient. The scanning skills including the ability to generate an adequate image are the most crucial parts in ultrasound and are best learned by hands on practice in very small supervised groups [7, 17, 32]. This limits the number of students and number of skills that can be taught – especially in a curriculum that is offered to all medical students [13]. The first task thus was to identify the most relevant skills to include in the curriculum. The skills proposed by the DEGUM were a good starting point since they sample a large variety of techniques, for example abdomen with B-mode sonography, lung with M-mode, sonography of the deep veins of the thigh with compression and colour Doppler; we added emergency echocardiography with colour doppler and duplex [10, 11]. In addition, these skills cover many common pathologies that have a high impact on the care of emergency patients and critically ill patients [4, 19, 23].

A second vital consideration related to the recruitment of instructors. We preferred to train student tutors over relying on faculty instructors to minimize group sizes and thus maximize the hands-on time for the individual student. Several different modalities have been proposed to train student tutors. Some proposals use internships in an ultrasound laboratory [1, 8, 21, 29], others employed course training [18, 29]. Both concepts have advantages and disadvantages: with an internship there is little control over the pathologies the student tutors encounter during their training time and the number of students that can be trained is limited by the capacity of the ultrasound laboratories. On the other hand, plenty opportunity for scanning practice can be offered. Using a course-based training enables full control over the contents, but the students have limited time to acquire the crucial practical scanning skills, and according to the analysis of Tarique et al., a trainig time of less than 2–3 weeks led to an inferior performance of the student tutors [29]. We therefore chose a hybrid concept and trained the students with a course to ensure that all the critical content is covered but added a four-week rotation through seven different ultrasound laboratories so that the students encounter considerable scanning practice and also have the chance to experience at least some of the pathologies in real life.

In our pilot implementation we found that the developed course was feasible and well accepted by the volunteer students with the student tutors rated as good as the faculty members. This is in accordance with previous findings of our group and other researchers [8, 16, 21]. The length of the course appears sufficient to teach the range of the selected skills, but is at the same time short enough so that the number of student tutor hours can be provided consistently, and the necessary pool of student tutors can be maintained within the capacity of our university hospital.

We did not evaluate the teaching outcome for tutees and the impact that the implementation of an ultrasound curriculum for all students has on the care of the patients. In this context, Fox et al. showed that more ultrasound examinations are performed in the emergency department, when medical students are present [15]. Future studies should research in detail how undergraduate ultrasound education of the students and student tutors affects their ability to care for patients.

## Conclusion

We developed a comprehensive ultrasound curriculum that (1) fulfills the requirements of the DEGUM and the NKLM, (2) samples important pathologies novice physicians may encounter (especially when caring for emergency and critically ill patients), and (3) can be implemented and sustained for all students. Future studies should investigate the potential impact of the curriculum on patient care.

## Data Availability

All data generated or analysed during this study are included in this published article.

## Abbreviations

DEGUM: Deutsche Gesellschaft für Ultraschall in der Medizin, German Ultrasound Society
eFAST: Extended Focused Assessment with Sonography for Trauma
FEEL: Focused Echocardiography in Emergency Life Support
NKLM: Nationaler Kompetenzbasierter Lernzielkatalog Medizin, Task Force National Competence-Based Learning Objectives for Undergraduate Medical Education
OSCE: Objective Structured Clinical Examination
